# Effect of Almond Skin Waste and Glycidyl Methacrylate on Mechanical and Color Properties of Poly(ε-caprolactone)/Poly(lactic acid) Blends

**DOI:** 10.3390/polym15041045

**Published:** 2023-02-20

**Authors:** Arantzazu Valdés, Franco Dominici, Elena Fortunati, Jose María Kenny, Alfonso Jiménez, María Carmen Garrigós

**Affiliations:** 1 Analytical Chemistry, Nutrition & Food Sciences Department, University of Alicante, P.O. Box 99, 03080 Alicante, Spain; 2 Department of Civil and Environmental Engineering, University of Perugia, 05100 Terni, Italy

**Keywords:** *Prunus amygdalus*, almond skin residues, PCL/PLA blends, glycidyl methacrylate, mechanical properties, color properties

## Abstract

Blending Poly(lactic acid) (PLA) and Poly(ε-caprolactone) (PCL) is a promising strategy to enhance the properties of biodegradable materials. However, these compounds are thermodynamically immiscible and, consequently, compatibilization is required during polymer blending. Reinforced biocomposites can be obtained by adding agricultural wastes generated by industries which are forced to consider waste treatment methods to prevent environmental concerns. Novel PCL/PLA blends were proposed based on the addition of 10 wt.% almond shell (AS) waste combined with 3 wt.% glycidyl methacrylate (GMA) as a compatibilizer. Different PCL-, PLA-, and PCL/PLA-based blends at different percentages (75:25, 50:50, 25:75, 15:85) added with GMA and AS were obtained. The color results highlighted the lower transparency and brownish tone of the studied formulations after the addition of AS. The addition of PCL provided a positive effect on PLA’s ductility due to its intrinsically higher flexibility. The combination of GMA and AS improved the mechanical properties of PCL, PLA, and 50:50 controls by reducing yield strength, yield strength at break, and elongation at break values. The 75:25_GMA_AS formulation showed a homogeneous visual appearance, low transparency, and desirable mechanical properties for rigid food packaging applications, reducing the final material cost through the revalorization of AS.

## 1. Introduction

Over the last few decades, the wide use of oil-based and nonbiodegradable plastic products has resulted in enormous amounts of macro and microplastics being accumulated in nature, resulting in a serious environmental pollution issue [1,2]. The global plastic production has increased from 2 million tons in 1950 to 359 million tons in 2019, and it is expected that the cumulative plastic production volume will reach 26 billion tons by the end of 2050 worldwide [3]. Specifically, the packaging industry produces a huge amount of plastic waste in some critical areas [4]. The best option for managing nonbiodegradable plastic waste is to replace uneconomical nonbiodegradable materials by recycling or reusing biodegradable polymers as they are environmentally friendly [5]. The global market for biodegradable polymers is expected to grow due to its high demand in a broad range of end-use industries across the globe, linked to stringent government regulations banning the use of synthetic plastics and the public perception of their negative effects.

Poly(lactic acid) (PLA) is a biodegradable and bio-based aliphatic polyester produced using the ring-opening polymerization of lactide [6,7]. It is one of the most popular examples of renewable polymers used for plastic products’ development. It has many potential advantages, such as excellent processability, nontoxic nature, thermal plasticity, similar mechanical properties as poly(ethylene terephthalate) (PET), and transparency [8]. PLA has a high modulus and tensile strength at break that is comparable to petroleum-based plastics [9]. It is also biodegradable in industrial composting and thermophilic biogas plants [3]. Currently, PLA is used in a variety of sectors, including food and beverages packaging in the form of cups, bottles, tea bags, or grocery bags; however, it is also used in mulch films, medical devices, and 3D printing applications [3]. However, the low elongation at break (~3%), brittleness, poor heat resistance, and hydrolytic instability have limited its use, primarily, to single-use disposable applications [10].

In contrast, poly(ε-caprolactone) (PCL) is a biodegradable and fossil-based thermoplastic semicrystalline aliphatic polyester obtained via the ring-opening polymerization of poly(caprolactone) and polycondensation of hydroxycarboxylic acid. It has important advantages, including high flexibility, good thermal stability with a low melting point, and biocompatibility. Currently, it is used for films, bags, biomedical applications, and 3D printing [11,12]. However, it has a lower modulus and exhibits a slower degradation rate when compared to PLA. To overcome such limitations, blending is one of the most effective approaches to combine the best attributes of both polymers. Remarkable research efforts have focused on PLA and PCL as the most important biodegradable materials [13,14,15,16,17] to be processed by traditional thermoplastic processing methods with a sustainable option for their disposal [13]. However, they are thermodynamically immiscible, resulting in a multiphase structure with poor dispersion and interfacial adhesion phase separation [9]. Up to now, various methods have been studied to improve the compatibility between phases in PLA/PCL blends, such as the addition of multi-block copolymers, reactive polymers, multi-functional polymeric compatibilizers, and fillers [18,19,20,21]. In this context, the use of natural reinforcements to improve the mechanical properties of biodegradable polymers for food packaging applications represents a promising method [22]. Goriparthi et al. [23] found that mixing PCL with PLA can improve mechanical properties, such as impact strength and damping capability, through the addition of a jute natural fiber (50 wt.%) to the formulation, without affecting other properties, such as biodegradability and biocompatibility. Rytlewski et al. [24] studied the reinforcement effect of a flax fiber addition (20 wt.%) to PCL/PLA blends, contributing to interphase adhesion enhancement and improving mechanical properties by increasing tensile strength and the elastic modulus. Priselac et al. [25] added coconut fibers (0–3 wt.%) to enhance the hardness of PCL/PLA blends, which is crucial to produce relief plates for embossing applications. On the other hand, different compatibilizers, such as nanoparticles, glycidyl methacrylate, dicumyl peroxide, and lysine triisocyanate, have been used in PLA/PCL blends for reducing the size of the dispersed phase and improving the toughness of the PLA matrix [6,7,26,27]. However, these compatibilizers are considered unsuitable environmentally friendly materials.

Almonds are a very important crop throughout the world’s temperate regions, with the worldwide almond production in 2021 being about 3.9 Mt [28]. The industrial processing of almonds starts with the removal of the almond skin (AS) by blanching, with this byproduct contributing to around 6.0–8.4 wt.% of the seeds [29]. Up to now, the valorization of these agricultural residues has not received enough attention, causing potential disposal problems since most of them are incinerated or dumped without control, causing severe environmental problems, such as air pollution, soil erosion, and a decrease in soil biological activity [30]. For these reasons, industries are forced to consider ways of treating or using them to not only prevent environmental concerns but also to provide farmers an extra income. Research on almond skin-reinforced composites in the literature is not very extensive. The addition of AS as a reinforcement agent into PCL by extrusion and injection molding has been previously reported [31,32], showing that PCL-based composites reinforced with AS at 10 wt.% loading had a clear improvement in mechanical properties as the result of an increase in the elastic modulus and hardness and a decrease in elongation at break, together with a high disintegration rate. Edward et al. [33] synthetized AS nanoparticles (0.25–1.0 wt.%) and studied their potential application in enhancing PLA film properties, showing an improvement on biodegradability and tensile strength of PLA added with 1.0 wt.% loading, as well as a reduction in water vapor permeability. The incorporation of low-cost AS residues into biodegradable polymers (such as PCL or PLA) is an attractive alternative to transform agricultural residues into useful industrial resources, with positive benefits in terms of the environment, energy savings, and economy. To the best of our knowledge, the development of almond-reinforced composites based on PCL/PLA blends has not been reported in the literature. Thus, the present study is focused on the preparation of novel PCL/PLA blends based on the addition of AS waste as a reinforcement agent, combined with glycidyl methacrylate (GMA) as a reactive compatibilizer for improving the phase interface between PLA and PCL [9]. Color and mechanical properties were studied to evaluate the effect of this compatibilization strategy on the developed blends.

## 2. Materials and Methods

### 2.1. Materials

Poly(ε-caprolactone) (PCL, CAPA^®^6800) commercial grade (pellets, Mn = 80,000 Da, density = 1.1 g cm^−3^) was kindly supplied by Perstorp Holding AB (Sweden). Poly(lactic acid) (Ingeo™ biopolymer 2003D, Mw = 193,000 Da, Mn = 114,000 Da, specific gravity = 1.24 g cm^−1^) was obtained from NatureWorks LLC. Almond skins, used as filler, were kindly supplied by “Almendras Llopis” (Alicante, Spain) as an industrial byproduct and were ground with a high-speed rotor mill (Ultra Centrifugal Mill ZM 200, RETSCH, Haan, Germany) equipped with a 1-mm sieve size. The obtained AS fraction was then dried in a laboratory oven at 100 °C for 24 h. Dimensions of AS particles consisted of a width average of 43 ± 12 mm and a length average of 107 ± 31 mm [32]. GMA was provided by Sigma–Aldrich (Madison, WI, USA).

### 2.2. Biocomposite Preparation

In this study, fifteen different formulations were obtained (Table 1) by combining several additives and/or PCL and PLA percentages. In these formulations, the separate and combined effect of the addition of GMA and AS on PCL, PLA, and PCL/PLA blends were studied. According to a previous work [9], GMA content was fixed at 3 wt.% based on the total mass of PLA and PCL. AS at 10 wt.% loading [32] was mixed with PLA or PCL before extrusion and, finally, GMA was added in the first minute of extrusion using a syringe to limit unnecessary losses.

Before extrusion, PLA and PCL pellets were dried overnight at 100 °C and 40 °C, respectively, to prevent polymer hydrolysis during processing. In addition, AS was dried for 2 h at 40 °C to eliminate moisture. Biocomposites were processed in a co-rotating twin-screw extruder (Xplore 5 & 15 Micro Compounder, DSM, Heerlen, The Netherlands). A rotating speed of 100 rpm for 3 min and a temperature profile of 170–180–190 °C in the three heating zones from the feeding section to die were used. After mixing, tensile dog-bone bars (ISO 527-2/5A) [34] were prepared by using a Micro Injection Molding Machine 10 cc (DSM). An appropriate pressure/time profile was used for injecting each type of sample, with an injection barrel temperature of 190 °C, while the mold’s temperature was set between 25 and 60 °C, depending on the sample.

### 2.3. Biocomposites Characterization

Color properties of biocomposites were determined, in triplicate, with a Konica CM3600d spectrophotometer (Konica Minolta Sensing Europe, Valencia, Spain) using the CIELAB color notation system (International Commission on Illumination). Three color parameters were determined: lightness (L*-axis), which changes from 0 or absolute black to 100 or perfect white; saturation (a*-axis), which changes from positive axis with red color to negative ones with green shares; and finally, the tone angle (b*-axis), which changes from a positive yellow axis to a negative blue one. The measured coordinates were used to calculate the whiteness index (WI), as obtained using Equation (1) [35]:

WI = 100 − [(100 – L*)^2^ +a*^2^ +b*^2^]^½^ (1)

For mechanical characterization, biocomposites were equilibrated for 48 h at 23 ± 1 °C and 50% RH before testing. Tensile tests were performed using a digital Lloyd instrument LR 30K with a cross-head speed of 5 mm min^−1^ and a load cell of 30 kN [23]. Dog-bone-shaped specimens (2 mm thick) [34,36] were tested according to the ISO 527 Standard [37]. Elongation at yield, yield strength, elongation at break, yield strength at break, and elastic modulus were calculated from the resulting stress–strain curves for all samples. Tests were carried out at room temperature and all values reported were the average of five measurements.

Hardness tests were carried out using a Shore D hardness tester (Instruments Brevetti Affri, Varese, Italy), following ASTM 2240-ISO/R 868 and DIN 53505 [38] Standards. Tests were performed in triplicate and average values were calculated.

### 2.4. Statistical Analysis

Statistical analysis of experimental data was performed by using one-way analysis of variance (ANOVA), expressed as means ± standard deviation. Differences between average values were assessed based on the Tukey test at a confidence level of 95% (*p* < 0.05). All statistical analyses were performed using StatGraphics Plus 5.0 software (Manugistics Inc., Rockville, MD, USA).

## 3. Results and Discussion

### 3.1. Color Properties

**Figure 1:**
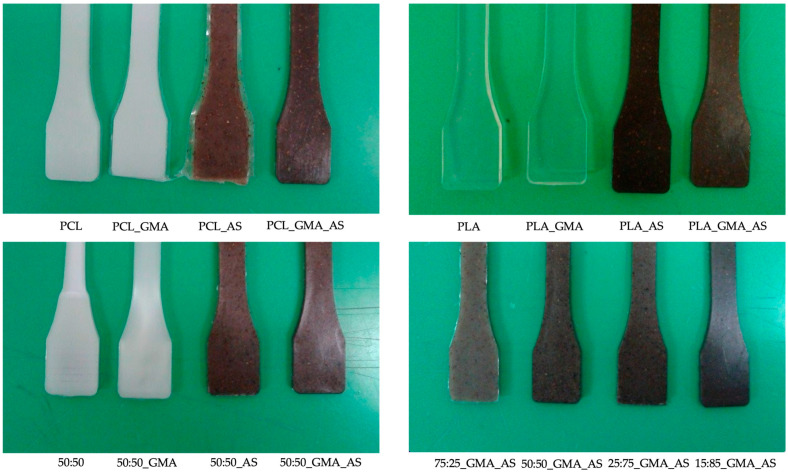
Visual appearance of the obtained biocomposites.

The visual appearance of the obtained biocomposites is shown in Figure 1. All samples showed a high visual homogeneity. However, the addition of the studied additives into PCL and PLA matrices induced some changes in color properties (Table 2). The obtained L* (lightness) and WI values for PLA_AS and PLA formulations ranged from 27.4 ± 2.6 to 83.2 ± 5.2 and from 27.1 ± 2.6 to 82.0 ± 4.7, respectively. PCL and PLA did not show statistically significant differences regarding these two parameters (*p* > 0.05). Moreover, the addition of GMA to both matrices did not produce a significant effect (*p* > 0.05) in color. However, a significant (*p* < 0.05) decrease in L* and WI values was found for biocomposites added with AS. Saturation (a*-axis) varied from −0.12 ± 0.02 for PLA_GMA to 6.18 ± 0.15 for 75:25_GMA_AS. When AS was not added, a* values ranged from −0.12 ± 0.02 for PLA_GMA to −0.98 ± 0.12 for 50:50, obtaining more negative values when the PCL matrix was used in contrast to PLA. After the addition of AS, a* results increased ranging from 3.99 ± 0.34 for PLA_AS to 6.18 ± 0.15 for 75:25_GMA_AS. Concerning the tone angle (b*-axis), it varied from 1.02 ± 0.35 for 50:50 to 8.34 ± 0.38 for 75:25_GMA_AS, showing a similar trend than that found for saturation after the AS addition, with the b* results increasing from 5.22 ± 0.20 for PLA_GMA_AS to 8.34 ± 0.38 for 75:25_GMA_AS. In conclusion, these results highlighted the lower transparency of the obtained formulations after the AS addition compared to PCL, PLA, or 50:50 blend controls, with and without adding GMA. The incorporation of AS contributed to intensify the biocomposites’ color due to the natural intrinsic brownish color of AS [35], being 75:25_GMA_AS, the formulation suggested as the most dark and opaque sample among the studied ones.

### 3.2. Mechanical and Shore D Hardness Tests

Five tensile parameters (Young’s modulus, yield strength, elongation at yield, yield strength at break, and elongation at break) and hardness values were evaluated in the studied biocomposites (Table 3). The addition of the additives to the PCL and PLA matrices as well as to the blends induced significant changes in the studied properties.

**Figure 2:**
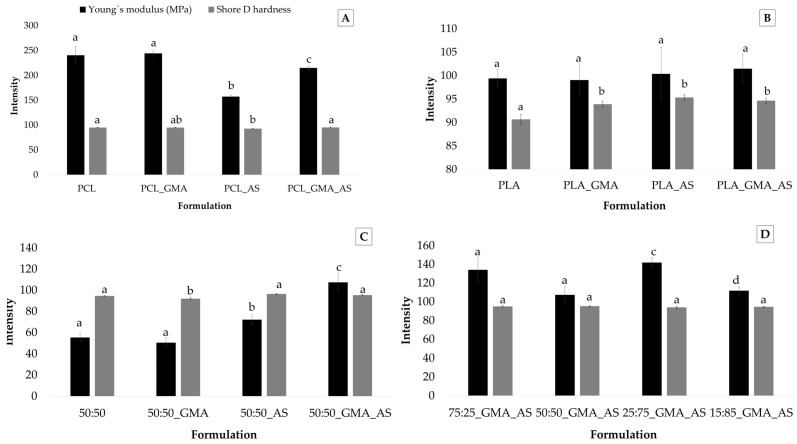
Young´s modulus (*n* = 5) and Shore D hardness (*n* = 3) values for PCL controls (**A**), PLA controls (**B**), PCL/PLA (50:50) controls (**C**), and PCL/PLA formulations (**D**). Mean ± SD. Different superscripts within the same parameter indicate statistically significant different values (*p* < 0.05).

Figure 2 shows the Young´s modulus and Shore D hardness values obtained for the PCL controls, PLA controls, PCL/PLA (50:50) controls, and PCL/PLA formulations. The hardness results showed some significant differences between samples, although they were not very noticeable. The obtained values were dependent on the matrix components, with results varying from 92.0 ± 1.0 for 50:50_GMA to 96.5 ± 0.5 for 50:50_AS (Table 3). The addition of GMA, AS, or both additives to PLA increased hardness values (Figure 2B), which could be attributed to the reinforcing effect of the additives and an increase in molecular interactions between components [4]. The addition of GMA and AS to different PCL/PLA ratios (Figure 2C) did not show statistically significant differences (*p* > 0.05) in hardness values except for 50:50_GMA, which showed the lowest hardness values of the studied formulations. In general, desirable hardness values were obtained for all samples intended for food packaging applications such as trays or containers.

Young’s modulus values ranged from 50 ± 9 MPa for 50:50_GMA to 244 ± 5 MPa for PCL_GMA (Table 3). As was expected, the PCL controls showed the highest values of all the studied samples [9,32], followed by PCL/PLA formulations, PLA controls, and, finally, 50:50 controls. Regarding the effect of the studied additives on PCL (Figure 2A), the addition of AS significantly (*p* < 0.05) reduced the elastic modulus of the polymer matrix from 240 ± 17 MPa for PCL to 157 ± 3 MPa for PCL_AS, whereas the addition of GMA significantly (*p* < 0.05) increased the Young´s modulus to 215 ± 4 MPa for PCL_GMA_AS. Thus, the addition of GMA enhanced the tensile properties of PCL_AS, which was probably due to a better fiber/matrix adhesion [23]. A similar effect was observed for the 50:50_GMA_AS formulation (Figure 2C). Therefore, the addition of GMA as a monomeric compatibilizer can be considered a successful strategy to improve the interfacial adhesion of the polymer phases in order to transfer the shear stress across the interface and, therefore, to improve the mechanical properties of the PCL and PCL/PLA blends reinforced with natural fibers. Sin and Han [9] studied the compatibilization of PCL and PLA blends added with GMA by following a melt-mixing and extrusion procedure. In this work, the decrease in the size of the dispersed PCL particles from the addition of GMA was related to the location of the added GMA at the interface between PLA and PCL phases, lowering the interfacial tension by wetting the interface, and, thereby, developing a finer dispersion of PCL domains. The glycidyl functional group of the epoxide has been suggested to react with carboxyl end groups (esterification) preferentially, followed by a reaction with hydroxyl groups (etherification) in the melt processing temperature range [39]. As a result, GMA enhanced interfacial adhesion between PCL and PLA, increasing the Young´s modulus values. Regarding the effect of the studied additives on PLA controls, no significant statistical differences (*p* > 0.05) were found between samples. However, the 25:75_GMA_AS sample showed the highest elastic modulus (142 ± 5 MPa) of all PCL/PLA formulations. These results could suggest the reinforcement effect of the combined addition of GMA and AS into the obtained biodegradable blends.

Regarding yield strength and yield strength at break, the lowest values were obtained for the PCL controls, whereas the highest ones were found for the PLA, PLA_GMA, and PLA_AS samples (Table 3). In fact, these formulations did not break under the experimental conditions used, suggesting the fragility of the PLA matrix. These results, together with the low elongation at yield (%) and elongation at break (%) values of the PLA formulations, were responsible for their high brittleness [40]. Regarding PCL controls, yield strength ranged from 15.9 ± 1.7 MPa for PCL to 17.7± 0.8 MPa for PCL_AS. A similar trend was observed for yield strength at break, since only the PCL_AS sample was broken under the experimental conditions (27.0 ± 1.6 MPa). Thus, the addition of AS to PCL decreased its ductility, which could be explained by considering that AS particles exert a resistance against the plastic deformation of the PCL matrix, which, in turn, restricts polymer chain elongation with an increase in the rigidity of the material due to the mobility in the amorphous region becoming increasingly restrained, as the filler is stiffer than the thermoplastic polymer. These results are in good agreement with those obtained when studying the mechanical behavior of PCL composites containing different lignocellulosic fillers, such as jute [23] and almond shell [31,32].

**Figure 3:**
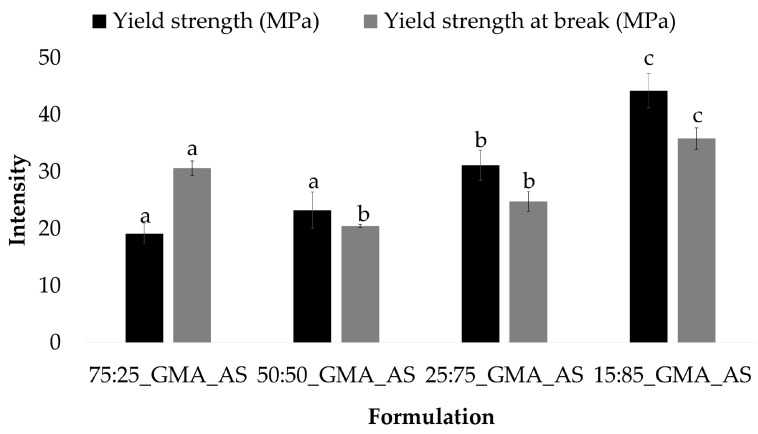
Yield strength (MPa) and yield strength at break (MPa) (*n* = 5) for PCL/PLA formulations at different concentrations. Mean ± SD. Different superscripts within the same parameter indicate statistically significant different values (*p* < 0.05).

PLA and 50:50 control samples also showed a similar behavior. In this formulation, the lowest values of the yield strength and yield strength at break were obtained after GMA and AS addition, with values of 45.6 ± 5.1 MPa and 38.6 ± 4.0 MPa for PLA_GMA_AS and 23.2 ± 3.2 MPa and 20.5 ± 0.3 MPa for 50:50_GMA_AS, respectively. All the other samples showed highest values with no statistically significant differences between them for each mechanical parameter (*p* > 0.05). Thus, the combination of GMA and AS improved the mechanical properties of PLA and 50:50 controls. Figure 3 shows the yield strength and yield strength at break values obtained for the studied formulations at different PCL/PLA concentrations. As can be seen, both parameters increased as the concentration of PCL decreased in the formulations, obtaining the highest values for 15:85_GMA_AS (44.2 ± 3.0 MPa and 35.9 ± 1.9 MPa, respectively). In conclusion, the addition of PCL induced a positive effect on PLA’s ductility due to its intrinsic higher flexibility [4,14,40].

**Figure 4:**
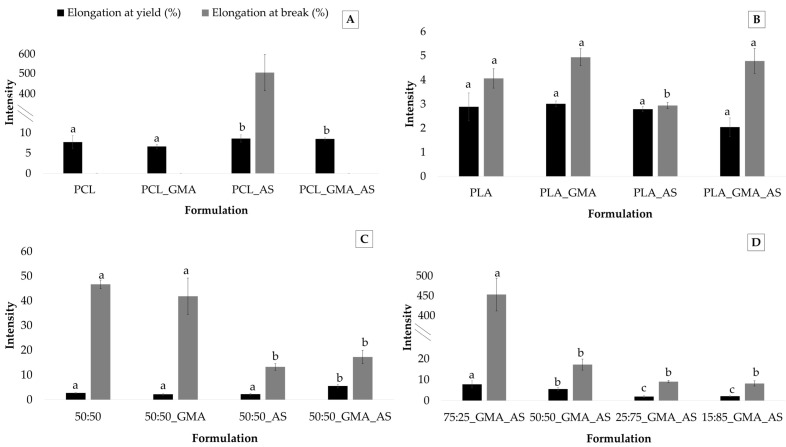
Elongation at yield (%) and elongation at break (%) (*n* = 5) for PCL controls (**A**), PLA controls (**B**), PCL/PLA (50:50) controls (**C**), and PCL/PLA formulations (**D**). Mean ± SD. Different superscripts within the same parameter indicate statistically significant different values (*p* < 0.05).

As was expected, elongation at yield and elongation at break parameters showed the lowest values for PLA-based samples, while the PCL matrices presented the highest values (Table 3). In fact, the PCL, PCL_GMA, and PCL_GMA_AS samples did not break under the tested experimental conditions. Figure 4 shows the elongation at yield (%) and elongation at break (%) values obtained for the PCL controls, PLA controls, PCL/PLA controls, and PCL/PLA formulations. Regarding PCL controls (Figure 4A), elongation at yield ranged from 6.6 ± 0.5 % to 8.6 ± 0.9 %. Higher nonstatistically significant (*p* > 0.05) values were found for PCL_AS and PCL_GMA_AS formulations. Only the PCL_AS sample broke during the tests, confirming the reinforcement effect of AS into PCL. This behavior was also observed after AS’s addition into PLA (Figure 4B) and the 50:50 blends (Figure 4C). Regarding PLA controls, although no statistically significant differences (*p* > 0.05) were found for elongation at yield, the lowest value of elongation at break was obtained for the PLA_AS sample (2.9 ± 0.2%). Figure 4C suggests that AS’s addition into the 50:50_AS and 50:50_GMA_AS samples decreased elongation at break values from 46.7 ± 1.8 % for 50:50 to 13.2 ± 1.3% for 50:50_AS and 14.3 ± 2.7% for 50:50_GMA_AS.

The plasticization effect of PCL to PLA is evidenced in Figure 4D. Concerning elongation at yield and elongation at break, an improvement in these parameters can be clearly observed, showing that the 75:25_GMA_AS sample the highest values of 7.8 ± 1.6 MPa and 450.8 ± 17.8 MPa, respectively. These results are in agreement with those obtained in other studies of mechanical parameters. Similar results were reported in other works by adding PCL into PLA [4,7,41], leading higher PCL contents to elongation at break values higher than 70% for compositions containing 22.5 wt.% PCL, which represented an increase in more than 715 % compared to neat PLA [14].

## 4. Conclusions

In this work, innovative PCL/PLA biocomposites added with 10 wt.% almond shell waste (AS) and 3 wt.% glycidyl methacrylate (GMA) as a reinforcement agent and compatibilizer, respectively, were obtained. The incorporation of AS contributed to intensify the color of the developed biocomposites due to the natural brownish color of the studied waste. The 75:25_GMA_AS formulation was suggested to be the darkest and most opaque sample among the studied ones. The color properties of the obtained formulations highlighted the potential of the PCL/PLA blends in the prevention of the oxidative deterioration of packaged foodstuff. Mechanical properties showed that the PCL addition provided a positive effect on PLA’s ductility due to its intrinsic higher flexibility. GMA enhanced interfacial adhesion between PCL and PLA, increasing the Young´s modulus values. AS particles exerted resistance against plastic deformation in the studied polymer matrices, which, in turn, restricted the polymer chain elongation, increasing the rigidity of materials. The combined addition of GMA and AS improved the mechanical properties of the PCL, PLA, and 50:50 controls by reducing the yield strength, yield strength at break, and elongation at break values. In general terms, the homogeneous visual appearance, low transparency, and mechanical properties of the 75:25_GMA_AS formulation would be promising for rigid packaging applications, such as trays or containers where transparency is not an issue. Finally, an additional advantage is the reduction in the packaging cost by adding and revalorizing AS, contributing to the circular economy concept.

## Figures and Tables

**Table 1:** Formulations obtained in this work.

Formulation	PCL (wt. %)	PLA (wt. %)	GMA (wt. %)	AS (wt. %)
PCL	100.00			
PCL_GMA	97.00		3	
PCL_AS	90.00			10
PCL_GMA_AS	87.00		3	10
PLA		100.00		
PLA_GMA		97.00	3	
PLA_AS		90.00		10
PLA_GMA_AS		87.00	3	10
50:50	50.00	50.00		
50:50_GMA	48.50	48.50	3	
50:50_AS	45.00	45.00		10
50:50_GMA_AS	43.50	43.50	3	10
75:25_GMA_AS	65.25	21.75	3	10
25:75_GMA_AS	43.50	43.50	3	10
15:85_GMA_AS	21.75	65.25	3	10

**Table 2:** Color parameters obtained for biocomposites. Mean ± SD (*n* = 3).

Formulation	L*	a*	b*	WI
PCL	79.9 ± 6.9 ^a^	-0.53 ± 0.16 ^a^	1.42 ± 0.45 ^a^	79.9 ± 6.9 ^a^
PCL_GMA	80.9 ± 0.6 ^a^	-0.58 ± 0.06 ^a^	1.75 ± 0.08 ^a^	80.8 ± 0.6 ^a^
PCL_AS	38.5 ± 4.3 ^b^	5.48 ± 0.49 ^bd^	6.33 ± 0.49 ^b^	38.0 ± 4.3 ^b^
PCL_GMA_AS	33.9 ± 3.2 ^b^	5.45 ± 0.65 ^bd^	6.99 ± 1.42 ^bc^	33.2 ± 3.3 ^b^
PLA	83.2 ± 5.2 ^a^	-0.15 ± 0.03 ^a^	6.18 ± 0.54 ^bc^	82.0 ± 4.7 ^a^
PLA_GMA	80.9 ± 4.7 ^a^	-0.12 ± 0.02 ^a^	4.37 ± 2.36 ^c^	80.2 ± 4.1 ^a^
PLA_AS	27.4 ± 2.6 ^b^	3.99 ± 0.34 ^c^	4.21 ± 0.16 ^bc^	27.1 ± 2.6 ^b^
PLA_GMA_AS	29.9 ± 2.7 ^b^	4.64 ± 0.39 ^bcd^	5.22 ± 0.20 ^bc^	29.6 ± 2.7 ^b^
50:50	78.8 ± 3.1 ^a^	-0.98 ± 0.12 ^a^	1.02 ± 0.35 ^a^	78.7 ± 3.1 ^a^
50:50_GMA	75.1 ± 2.1 ^a^	-0.45 ± 0.39 ^a^	2.53 ± 0.40 ^ac^	74.9 ± 2.1 ^a^
50:50_AS	35.1 ± 0.9 ^b^	5.43 ± 0.18 ^bd^	6.90 ± 0.13 ^b^	34.6 ± 0.9 ^b^
50:50_GMA_AS	35.2 ± 0.7 ^b^	5.98 ± 0.17 ^d^	7.62 ± 0.14 ^b^	34.5 ± 0.7 ^b^
75:25_GMA_AS	40.0 ± 1.4 ^b^	6.18 ± 0.15 ^d^	8.34 ± 0.38 ^b^	39.1 ± 1.3 ^b^
25:75_GMA_AS	34.8 ± 0.2 ^b^	5.49 ± 0.05 ^bd^	7.19 ± 0.05 ^b^	34.2 ± 0.2 ^b^
15:85_GMA_AS	30.2 ± 0.6 ^b^	5.05 ± 0.22 ^bd^	5.20 ± 0.25 ^bc^	29.8 ± 0.6 ^b^

Different superscripts within the same column and parameter indicate statistically significant different values (*p* < 0.05).

**Table 3:** Mechanical (*n* = 5) and D shore (*n* = 3) results obtained for biocomposites. Mean ± SD.

Formulation	Young Modulus (MPa)	Yield Strength (MPa)	Elongation at Yield (%)	Yield Strength at Break (MPa)	Elongation at Break (%)	Shore D Hardness
PCL	240 ± 17 ^a^	15.9 ± 1.7 ^a^	7.7 ± 1.6 ^ac^	Not break	Not break	95.0 ± 0.5 ^a^
PCL_GMA	244 ± 5 ^a^	17.1 ± 0.2 ^a^	6.6 ± 0.5 ^ad^	Not break	Not break	94.8 ± 0.3 ^ac^
PCL_AS	157 ± 3 ^b^	17.7 ± 0.8 ^a^	8.6 ± 0.9 ^c^	27.0 ± 1.6 ^ae^	534.8 ± 19.4 ^a^	92.7 ± 0.6 ^c^
PCL_GMA_AS	215 ± 4 ^c^	16.8 ± 0.3 ^a^	8.5 ± 0.3 ^c^	Not break	Not break	95.2 ± 0.8 ^a^
PLA	99 ± 2 ^d^	71.0 ± 4.9 ^b^	2.9 ± 0.6 ^b^	58.5 ± 5.0 ^b^	4.1 ± 0.4 ^b^	90.7 ± 1.0 ^b^
PLA_GMA	99 ± 3 ^d^	69.4 ± 3.8 ^b^	3.0 ± 0.2 ^b^	59.5 ± 2.3 ^b^	4.9 ± 0.3 ^b^	93.9 ± 0.7 ^ac^
PLA_AS	100 ± 6 ^d^	61.5 ± 2.0 ^b^	2.8 ± 0.2 ^b^	59.6 ± 2.4 ^b^	2.9 ± 0.2 ^e^	95.3 ± 0.6 ^ac^
PLA_GMA_AS	101 ± 3 ^d^	45.6 ± 5.1 ^c^	2.0 ± 0.4 ^b^	38.6 ± 4.0 ^c^	4.8 ± 0.5 ^b^	94.7 ± 0.6 ^ac^
50:50	55 ± 4 ^e^	36.1 ± 0.6 ^d^	2.8 ± 0.2 ^b^	27.0 ± 0.2 ^a^	46.7 ± 1.8 ^c^	94.6 ± 0.4 ^ac^
50:50_GMA	50 ± 9 ^e^	38.4 ± 7.9 ^cd^	2.2 ± 0.3 ^b^	27.5 ± 1.1 ^a^	41.9 ± 7.4 ^c^	92.0 ± 1.0 ^d^
50:50_AS	72 ± 5 ^f^	34.4 ± 2.3 ^d^	2.3 ± 0.3 ^b^	28.0 ± 1.4 ^a^	13.2 ± 1.3 ^d^	96.5 ± 0.5 ^a^
50:50_GMA_AS	107 ± 5 ^e^	23.2 ± 3.2 ^a^	5.5 ± 0.7 ^d^	20.5 ± 0.3 ^d^	14.3 ± 2.7 ^d^	95.3 ± 0.6 ^a^
75:25_GMA_AS	134 ± 13 ^e^	19.1 ± 1.8 ^a^	7.8 ± 1.6 ^ac^	30.6 ± 1.3 ^a^	450.8 ± 17.8 ^f^	95.0 ± 1.0 ^a^
25:75_GMA_AS	142 ± 5 ^b^	31.1 ± 2.7 ^d^	2.0 ± 0.4 ^b^	24.8 ± 1.7 ^a^	9.1 ± 0.5 ^d^	94.0 ± 1.3 ^acd^
15:85_GMA_AS	112 ± 5 ^d^	44.2 ± 3.0 ^c^	2.1 ± 0.2 ^b^	35.9 ± 1.9 ^e^	8.2 ± 1.2 ^d^	94.7 ± 0.6 ^ac^

Different superscripts within the same column and parameter indicate statistically significant different values (*p* < 0.05).

## Data Availability

The data presented in this study are available on request from the corresponding author.
